# Thromboelastography-Guided Anesthetic Management for Cesarean Section in a Patient With Antiphospholipid Syndrome: A Case Report

**DOI:** 10.7759/cureus.89829

**Published:** 2025-08-11

**Authors:** Miho Wakabayashi, Yasuyuki Tokinaga, Tomoyuki Kawamata

**Affiliations:** 1 Faculty of Medicine, Department of Anesthesiology, Wakayama Medical University, Wakayama, JPN

**Keywords:** antiphospholipid syndrome, emergency cesarean section, residual heparin effect, subcutaneous unfractionated heparin, thromboelastography

## Abstract

In pregnant women with antiphospholipid syndrome (APS) receiving subcutaneous unfractionated heparin, activated partial thromboplastin time (aPTT) cannot reliably evaluate anticoagulant effects due to interference from antiphospholipid antibodies. Thromboelastography (TEG) can assess both heparin effects and overall coagulation status.

A pregnant woman with APS on aspirin and subcutaneous unfractionated heparin developed genital bleeding, necessitating an emergency cesarean section. The interval since the last heparin dose was borderline for safe neuraxial anesthesia. TEG was performed to evaluate coagulation status and revealed impaired coagulation, likely due to residual heparin effect and reduced coagulation factors from bleeding. General anesthesia was selected. Surgery proceeded uneventfully under general anesthesia with intraoperative transfusion of fresh frozen plasma. Hemostasis was maintained perioperatively.

TEG provided critical information for anesthesia decision-making and intraoperative management in a pregnant patient with APS receiving heparin, helping guide safe and effective clinical care.

## Introduction

Antiphospholipid syndrome (APS) is a systemic autoimmune disease characterized by thrombotic events. During pregnancy, thromboprophylaxis with low-dose aspirin and unfractionated heparin is generally recommended to reduce the risk of maternal and fetal complications [1]. The anticoagulant effect of unfractionated heparin is monitored by measuring activated thromboplastin time (aPTT). However, in patients with APS, aPTT is often prolonged even in the absence of actual coagulation abnormalities due to interference from antiphospholipid antibodies with phospholipid-dependent reactions [2]. As a result, aPTT is likely to provide an unreliable assessment of heparin’s anticoagulant effect in this population.

Thromboelastography (TEG) is a simple, point-of-care test that provides a comprehensive assessment of the coagulation-fibrinolysis system. TEG can evaluate the effect of residual heparin by comparing the reaction time (R) values from citrated kaolin (CK) and CK with heparinase (CKH) thromboelastography, which cannot be detected by aPTT [3]. TEG has been reported to show normal values even in patients with APS whose aPTT is prolonged due to the presence of antiphospholipid antibodies [4,5].

Here, we report a case in which thromboelastography (TEG6s®, Haemonetics, Boston, MA, USA) was employed to assess the anticoagulant effect of unfractionated heparin in a pregnant woman with APS for the purpose of determining the anesthetic strategy for cesarean section. Written informed consent was obtained from the patient for publication of this case report and accompanying images.

## Case presentation

A 25-year-old Japanese woman with mixed connective tissue disease and APS was treated with prednisolone, tacrolimus, and azathioprine. The patient had been receiving low-dose aspirin (81 mg daily) and subcutaneous injections (SC) of unfractionated heparin calcium (10,000 units/day in two divided doses) during pregnancy, which are typical regimens for pregnant women with APS as reported in previous studies [2]. Her pregnancy course had been uneventful, but an elective cesarean section was scheduled at 37 weeks and three days of gestation due to her short stature (height: 139 cm, patient's pre-pregnancy weight: 45 kg, and preoperative weight: 48.8 kg) and cephalopelvic disproportion. At 37 weeks and two days of gestation, she experienced genital bleeding (estimated amount: 500 mL) without evidence of non-reassuring fetal status, which prompted an immediate emergency cesarean section to ensure maternal and fetal safety. Her vital signs were stable, conscious, BP 120/83 mmHg, and HR 98 bpm on admission. While administration of aspirin had been discontinued nine days prior to the day of the emergency surgery, 5000 units of SC heparin calcium had been administered approximately eight hours prior to the surgery. She had ingested solid foods one hour prior to presentation; therefore, neuraxial anesthesia was initially preferred over general anesthesia in consideration of the risk of aspiration. However, because the interval since the last heparin dose was shorter than the 8-10 hours recommended by the Japanese guideline [6] (although within the 4-6 hours recommended by the American Society of Regional Anesthesia and Pain Medicine (ASRA) guideline [7]), we performed TEG to assess residual heparin activity before determining the anesthetic method. If the interval had exceeded 10 hours, we would have proceeded with neuraxial anesthesia without performing TEG.

Preoperative laboratory data are summarized in Table 1. The hemoglobin value was one week before surgery: 9.8 g/dL; before surgery: 10.2 g/dL. The pre-heparin aPTT is 50.5 seconds, which is prolonged beyond the reference range, a finding commonly observed in patients with antiphospholipid syndrome due to interference from antibodies. PT-INR remained within the normal range. Fibrinogen was elevated, likely reflecting an acute-phase response. Other parameters such as hemoglobin, platelet count, and prothrombin time were also within or near the normal range. Given the known limitations of aPTT in this context, we determined that it did not accurately reflect her actual coagulation status.

**Table 1:** Preoperative laboratory findings prior to emergency cesarean section All results were obtained within one hour before surgery. The prolonged aPTT is likely related to the presence of antiphospholipid antibodies. Elevated fibrinogen may reflect an acute-phase response. PT-INR remained within the normal range. aPTT: Activated partial thromboplastin time PT: Prothrombin time

Test (Unit)	Value	Reference Range
Hemoglobin (g/dL)	10.2	11.5-15.0
Platelet count (/µL)	163,000	150,000-350,000
aPTT (seconds)	43.3	26-38
PT (seconds)	10.6	10-13
PT-INR	0.97	0.9-1.1
Fibrinogen (mg/dL)	480	160-350

**Figure 1:**
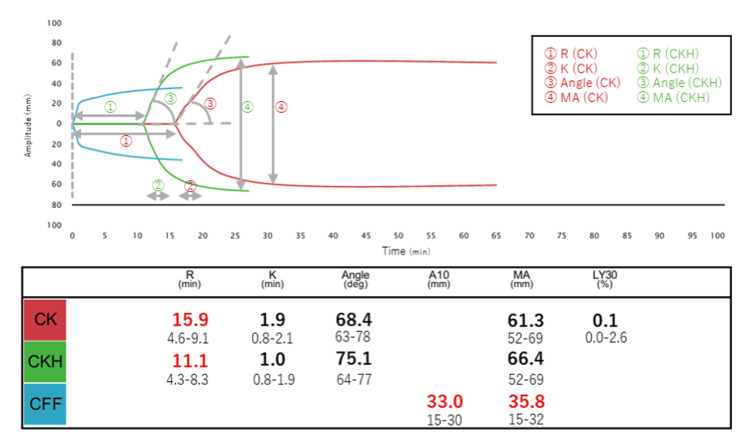
Thromboelastography findings in this case The citrated kaolin (CK) assay, which utilizes the coagulation activator kaolin, primarily activates the intrinsic pathway, revealing hemostatic characteristics. The CK with heparinase (CKH) assay, which contains kaolin and a heparin-neutralizing enzyme, enables the assessment of hemostatic characteristics without the influence of heparin. The citrated functional fibrinogen (CFF) assay isolates the function of fibrinogen, which contributes to the strength of the clot. R (min) is the time until the thrombus amplitude reaches 2 mm. K (min) is the time from the thrombus amplitude reaching 2 mm until the amplitude reaches 20 mm. The angle is the slope of a tangent line from the tracing at the midpoint between the R and K times. A10 is the thrombus amplitude at 10 min, and MA is the absolute thrombus strength. LY30 (%) represents the percentage of fibrinolysis at 30 min after max amplitude. Overview of TEG6s^®^ data: The values in red indicate that they are higher than the basal value. R in the CK assay was prolonged more than that in the CKH assay. R in the CKH assay was also prolonged. A10 and MA in the CFF assay were slightly higher than normal. Basal values are shown below each measured value.

To more accurately evaluate her coagulant status, we performed TEG using the TEG6s® (Figure 1). The reaction time (R) in the citrated kaolin (CK) assay was prolonged to 15.9 minutes (min) (reference range: 4.6-9.1 min), and R in the CK with heparinase (CKH) assay was also prolonged to 11.1 min (reference range: 4.3-8.3 min). The greater prolongation in the CK assay compared to the CKH assay suggested a residual anticoagulant effect of SC heparin. The prolonged R value in the CKH assay was presumed to be due to consumption of coagulation factors associated with active bleeding. Despite this, fibrinogen levels were preserved, and both the A10 and maximum amplitude (MA) values in the functional fibrinogen (CFF) assay were slightly elevated above normal. These findings indicated a residual heparin effect and mild consumption coagulopathy, rather than interference from antiphospholipid antibodies, as the cause of the prolonged aPTT. Platelet function and fibrinolysis parameters were within normal limits.

Based on the results of the TEG findings, we elected to proceed with general anesthesia rather than neuraxial block. Following preoxygenation, rapid sequence induction was performed with propofol 2 mg/kg, rocuronium 1.2 mg/kg, and remifentanil 0.1 mg. Endotracheal intubation was carried out after confirming a bispectral index below 60 and a train-of-four count of 0. The baby was delivered four minutes after the skin incision, with Apgar scores of 3 at 1 minute and 9 at 5 minutes. Four units of fresh frozen plasma were transfused intraoperatively. Hemostasis was satisfactory, and total blood loss, including amniotic fluid, was 650 mL. Red blood cell transfusion was not required. The newborn weighed 2271 g and was admitted to the neonatal intensive care unit for scheduled observation. No maternal complications were observed.

## Discussion

TEG evaluates the coagulation activation rate, interaction of coagulation factors and platelets, clot strength, and fibrinolytic processes using whole blood under conditions that approximate in vivo physiological responses [8]. It has been used to assess pregnancy-related changes in coagulation [9] and to support the management of obstetric complications such as HELLP syndrome [10], thrombophilia after cesarean section [11], and obstetric hemorrhage, for which European guidelines recommend real-time TEG monitoring [12].

In this case, conventional coagulation tests, including aPTT, suggested a prolonged clotting time, which could be influenced by the presence of lupus anticoagulant in antiphospholipid syndrome. However, thromboelastography (TEG) provided complementary information by directly assessing clot formation and strength. The TEG performed with kaolin (CK) demonstrated a prolonged reaction time, while the kaolin with heparinase (CKH) channel showed normalization of clotting parameters. This simultaneous comparison indicated the presence of a residual heparin effect, confirming anticoagulant activity that might not be fully characterized by standard laboratory tests.

In the present case, aPTT was prolonged, but this finding can be influenced by antiphospholipid antibodies and does not reliably reflect coagulation status in vivo. Because the interval from the last heparin dose was only 8 hours, at the lower limit of the Japanese guideline (8-10 hours), which was prioritized in this case, we used TEG to evaluate residual heparin activity. Although the ASRA guideline suggests a shorter interval of 4-6 hours, the Japanese recommendation was considered more relevant in our clinical context, and TEG was used to confirm safety at this borderline timing. TEG revealed a prolonged CK-R compared with CKH-R, consistent with residual heparin, as supported by previous studies [3,13] demonstrating that this parameter correlates with plasma heparin concentration.

Based on these results, and because residual heparin activity was confirmed, general anesthesia was selected rather than neuraxial anesthesia, despite the patient’s stable vital signs and relatively limited blood loss (500 g). If more than 10 hours had elapsed since the last heparin dose, we would have proceeded with neuraxial anesthesia without TEG. This case highlights the value of TEG not only in confirming residual heparin but also in guiding safe anesthetic decision-making in patients with APS.

Current recommendations for neuraxial anesthesia after heparin therapy are based largely on the half-life of heparin [6,7] and do not account for individual variability. Our case suggests that individualized assessment with TEG may be useful to complement guideline-based timing, particularly in high-risk patients such as those with APS. In particular, TEG was valuable in this case because it allowed assessment of the residual heparin effect, which cannot be reliably evaluated by aPTT in patients with APS.

This case also has limitations, including the lack of follow-up TEG data after heparin cessation, which could have provided additional confirmation of heparin’s contribution to the initial abnormal findings.

## Conclusions

There have been no reports of TEG in pregnant women with APS who required emergency cesarean section during antithrombotic therapy. In the present case, use of a bedside TEG6s^®^ analyzer was useful to obtain detailed information on the coagulation-fibrinolysis system that could not be obtained from a routine coagulation test. This case also highlights that TEG may complement guideline-based timing of neuraxial anesthesia by allowing individualized assessment of coagulation function in patients receiving heparin.
